# Design and Implementation of Fast Fault Detection in Cloud Infrastructure for Containerized IoT Services

**DOI:** 10.3390/s20164592

**Published:** 2020-08-16

**Authors:** Hyunsik Yang, Younghan Kim

**Affiliations:** School of Electronic Engineering, Soongsil University, Seoul 06978, Korea; yangun@dcn.ssu.ac.kr

**Keywords:** fault detection, container, Internet-of-Things (IoT) cloud, edge cloud

## Abstract

The container-based cloud is used in various service infrastructures as it is lighter and more portable than a virtual machine (VM)-based infrastructure and is configurable in both bare-metal and VM environments. The Internet-of-Things (IoT) cloud-computing infrastructure is also evolving from a VM-based to a container-based infrastructure. In IoT clouds, the service availability of the cloud infrastructure is more important for mission-critical IoT services, such as real-time health monitoring, vehicle-to-vehicle (V2V) communication, and industrial IoT, than for general computing services. However, in the container environment that runs on a VM, the current fault detection method only considers the container’s infra, thus limiting the level of availability necessary for the performance of mission-critical IoT cloud services. Therefore, in a container environment running on a VM, fault detection and recovery methods that consider both the VM and container levels are necessary. In this study, we analyze the fault-detection architecture in a container environment and designed and implemented a Fast Fault Detection Manager (FFDM) architecture using OpenStack and Kubernetes for realizing fast fault detection. Through performance measurements, we verified that the FFDM can improve the fault detection time by more than three times over the existing method.

## 1. Introduction

Container-based clouds are used in various service infrastructures because they are lighter and more portable than a virtual machine (VM)-based infrastructure and are configurable in both bare-metal and VM environments [1]. The internet-of-things (IoT) cloud-computing infrastructure is also evolving from a VM-based to a container-based infrastructure [2,3,4,5]. In IoT clouds, the service availability of the cloud infrastructure is more important for mission-critical IoT services, such as real-time health monitoring, vehicle-to-vehicle (V2V) communication, and industrial IoT, than it is for general computing services [6,7,8,9,10,11]. Service availability can usually be improved by using various fault detection and recovery methods. Fast fault detection is essential for quick recovery from faults [12,13,14,15,16]. These research works were conducted to improve fault detection and availability based on VM-based infrastructure, but with the industry shift to a container-based cloud environment, research has started towards improving fault detection and availability in container environments.

The container-based cloud infrastructure can be deployed on the VM or bare metal server directly, and it is usually managed by Kubernetes, which is a container orchestrator. Hence, it is necessary to appropriately set the Kubernetes parameters related to fault detection and recovery to meet the requirements of mission-critical IoT services [17]. To study fault recovery in container infrastructure, References [18,19] measured and analyzed the fault detection and recovery performance under various conditions in the Kubernetes environment. First, in Reference [18], the fault detection and recovery function were tested using the basic Kubernetes function when the case of node failure and application fault occurred. However, the authors of Reference [18] measured and presented only fault detection and recovery based on default parameters. In addition, since the focus is only on functional tests for failure recovery at the application level, the improvement of the node’s fault detection method is not considered.

In Reference [19], based on the result of Reference [18], when a node fault occurs, the fault detection time and the fault recovery time were measured. However, in the case of node fault, the performance was measured considering only the fault detection time (reaction time) at the application level, such as load balancer. In addition, although some parameters related to fault detection in Kubernetes have been modified, a method for improving the fault detection method has not been considered.

Abdollahi et al. [20] proposed a method of ensuring availability based on appropriate storage management. The service was configured as a redundancy model, and the architecture was proposed to share data via Persistent Volume (PV). A state controller was proposed on the existing architecture to configure two pods as an active and standby model and shared one PV to share the data was designed. As a perspective of availability, the proposal in Reference [20] is also considered, but further research is needed on how to guarantee availability starting from node fault to reduce the service outage, due to node fault.

These studies considered only the performance measurement in the existing environment and the recovery of faults at the service level. Therefore, in order to reduce the fault detection time of a node, a method for quickly detecting a node fault is required. Especially, container environments running on VMs require a method of the fault detection and recovery that considers both VM and container levels. Through this, it is possible to optimize fault detection time to ensure the level of availability required by a mission-critical IoT cloud. However, these studies do not describe how faults can be detected quickly enough for mission-critical IoT services running on the VM.

In this study, we analyze the fault-detection architecture in a container environment and design and implement an integrated fault-management architecture for realizing fast fault detection. We consider the addition of a fault detection system at the VM level and its integration with the container fault detection system, as necessary, to plug the gap in swift detection of faults occurring, due to failures in the container environment infrastructure running on the VM. We design and implement Fast Fault Detection Manager (FFDM) using OpenStack and Kubernetes. FFDM is a component for interworking the functions of the VM orchestrator and Container orchestrator, and it provides an automated monitoring function for quick fault detection and recovery and the requisite function to deliver VM fault information to the container orchestrator directly. Further, we show that the proposed architecture can improve both the fault-detection speed and fault-recovery time using measurements from the implementation. In summary, the contributions of this study are as follows:

(a) We analyze the fault-detection architecture in a container environment and highlight its limitations
(b) We design and implement Fast Fault Detection Manager (FFDM) using OpenStack and Kubernetes; an integrated architecture which provides an automated monitoring function for quick fault detection and recovery.
(c) Design and implementation of an architecture for fault information delivery according to the monitoring results.
(d) We evaluate the performance of our proposed architecture against the current state of the art approaches and show that it can improve both the fault-detection speed and fault-recovery time.

The rest of this study is organized as follows. In Section 2, the fault-detection procedure of the container infrastructure is analyzed. In Section 3, the fast-fault-detection architecture is described; and subsequently, in Section 4, we present an analysis of the experimental results. Section 5 concludes the study and offers directions for possible future works.

## 2. State of the Art: Fault Detection and Recovery Mechanisms in Container Infrastructure

We present common fault-management methods for container environments [17] in Table 1.

Kubernetes supports three probe methods for checking the status of applications, namely, TCP, HTTP, and command. The probes can be divided into two categories, depending on whether they check for liveness or readiness. Liveness probes provide the ability to periodically check the current application status. When a problem is confirmed, the application is restarted by the Kubelet. Readiness probes are used to check the status of the pod after migrating, restarting, or reconfiguring the pod. If the pod is not ready to perform normal service, Kubernetes removes the IP address of the pod from all the services to prevent faults in the service. In addition to these probes, Kubernetes uses external monitoring tools, such as Prometheus, to compensate for faults that may be missed [21].

Containers run in pods that are deployed on nodes (physical or logical servers). The node status is periodically updated by the Kubelet running on the worker nodes to the Kubernetes master node. There are four parameters related to this process, as listed in Table 2.

**Figure 1:**
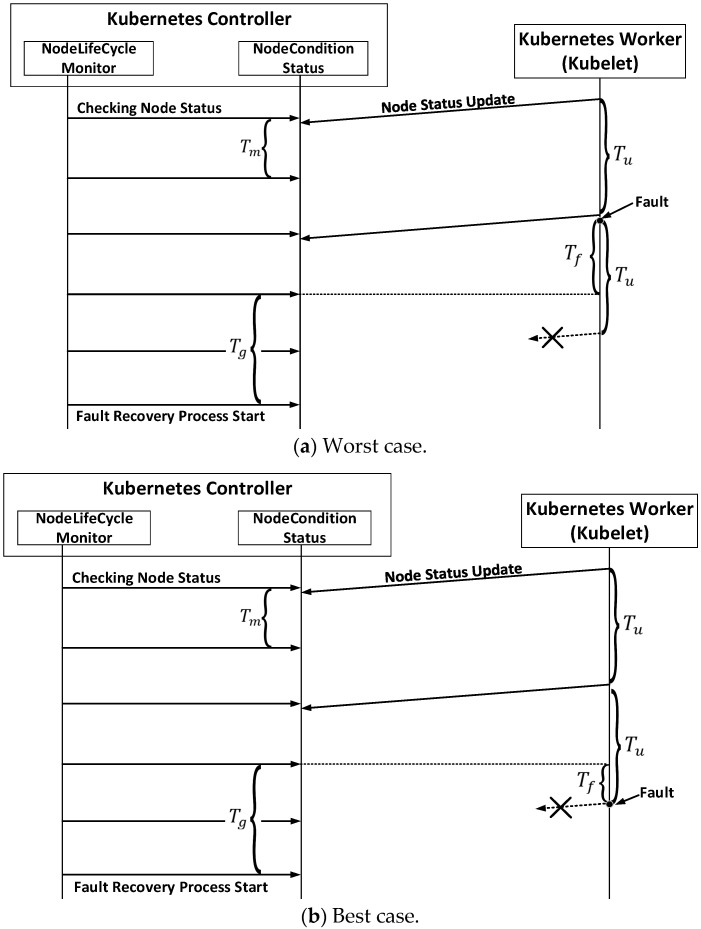
Timing diagrams of the fault detection process.

**Figure 2:**
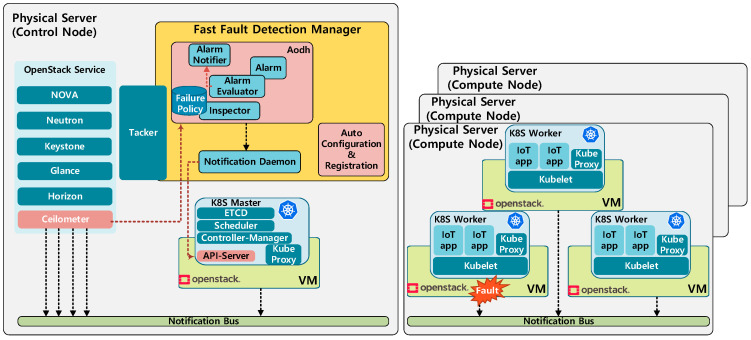
Proposed cloud architecture for fast fault detection.

As shown in Figure 1 and Figure 2, the node-status-update-frequency parameter is used to set the frequency at which the Kubelet sends the status of the node to the Kubernetes master node. The node-monitor-period parameter sets the monitoring time interval for the Kubernetes master node to check the status of the nodes updated by the Kubelet. The third parameter, node-monitor-grace-period, controls the length of time that the master node defers its decision to change the state of a node if its updated information does not confirm a normal node status. The pod-eviction-timeout is the duration of time to wait before removing a node that has been determined to be defective. The default duration is 5 min, after which the pod migration begins.

Adjusting the values of these parameters can reduce the fault detection and recovery time. Several studies have been conducted on the fault detection and repair processes in Kubernetes.

The authors of Reference [18] tested the basic fault detection and recovery capabilities of Kubernetes. However, they only used the default parameter values in their measurements. Based on the same architecture as [18], the optimization of failure detection and recovery was studied in Reference [19], but only pod- or container-level processes were considered except for node-level fault detection.

In Reference [20], a method was proposed to improve the availability of stateful services through common storage for the active and standby pods, but node-level fault handling was not considered.

In this study, we first analyzed the Kubernetes fault detection times when node failures occurred using the parameters defined in Table 2. To do that, we added a new parameter ‘$T_{f}$’ to express the time interval between the fault occurrence point and the grace period endpoint. All the numerical parameters in Kubernetes are presented in s. The methodology for setting the parameters is as follows. The node-monitor-grace-period ‘$T_{g}^{\prime }$ was set to a multiple of $T_{u}$, i.e., $T_{g}=M\times T_{u},\;M\geq 1,$ with the default value of 4 for M. This means that Kubernetes provides a grace time of 4 Kubelet cycles for fault determination. $T_{m}$ should be set to a smaller value than $T_{u}$. If $T_{m}$ is larger than $T_{u}$, node status reporting messages from the Kubelet will not be checked by the node monitor over the $T_{m}$ period, which will increase the fault detection time. The default value of $T_{u}$ was set as twice of $T_{m}$.

Figure 1 presents the worst- and best-case scenarios for fault detection. In this figure, we assumed that $T_{u}$ is twice the value of $T_{m}$, and $T_{u}$ and $T_{g}$ have the same value. The worst case for fault detection is when a fault occurs at the node shortly after the Kubelet has reported that the node is healthy. In this case, we obtain the worst-case fault-detection time of

(1) $$T_{fd\text{-}kube}^{worst}=T_{g}+T_{f}$$

The best case for fault detection is when the node fails just before the Kubelet reports the node status. In this case, the timer of duration $T_{g}$ started at the previous checkpoint has just expired, and as a result, it is determined that some node defects have occurred. Consequently, the fault detection time becomes shorter than $T_{g}$ and is given by

(2) $$T_{fd\text{-}kube}^{Best}=T_{g}-T_{f},\;\left(0<T_{f}<T_{m}\right)$$

From Equations (1) and (2), the range of the fault-detection time is

(3) $$T_{g}-T_{f}\leq T_{fd\text{-}kube}\leq T_{g}+T_{f}\;\left(0<T_{f}<T_{m}\right)$$

As shown in Figure 1, the existing method has a difference in detection time, depending on when the fault occurred. In addition, due to the correlation of parameters described above, and then the procedure necessary to determine the fault, there is a limit to improve the fault detection time in the existing architecture. The above-mentioned papers also presented the performance measurement results of the existing fault detection method in the VM-based container structure, but the improvement of the fault detection time in the container environment deployed in the VM was not considered. To improve this, a way to quickly detect fault from a node and forward it directly to the Kubernetes Controller should be considered.

## 3. Proposed Architecture

In this section, we describe the proposed fault-detection architecture for containers deployed on VMs. Instead of detecting VM faults at the container level, we use VM-level fault detection and notify the container level of the detected faults directly. To this end, we designed a fast fault detection manager (FFDM) that automatically registers information for VM failure monitoring and forwards the failure information delivered to the VM orchestrator to Kubernetes [17,22].

**Figure 3:**
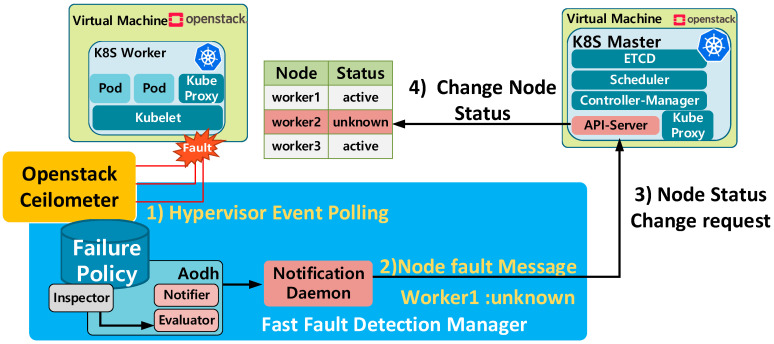
Fast-fault-detection manager.

As depicted in Figure 2, VMs are orchestrated by the OpenStack control node and operate as nodes in a container cluster. The cluster is managed by Kubernetes and consists of a master node in charge of controlling the containers and worker nodes on which the containers are deployed. As shown in Figure 3, the fast-fault-detection technology defined in the OPNFV project [23] is applied to this IoT cloud. For fast detection of VM faults, the FFDM registers fault alert policies, such as maximum resource utilization and VM down, with the VM-level fault monitoring and reporting components, such as Ceilometer and Aodh [24,25]. The ceilometer periodically collects events generated by the hypervisor to check the VM status, and when a fault occurs, an alarm is generated through Aodh. The polling cycle of ceilometer, which gets the information from the hypervisor that manages virtual resources, is 300 ms; and when an alarm occurs, a message is sent to the VM management function immediately. In this paper, the notification daemon checks the fault information and is designed to transmit the node status change message to the Kubernetes Master. When sending fault information to Kubernetes Master, it converts the information according to the Kubernetes format and sends it to the Kubernetes master through REST API, and this process is only necessary for fault nodes.

**Figure 4:**
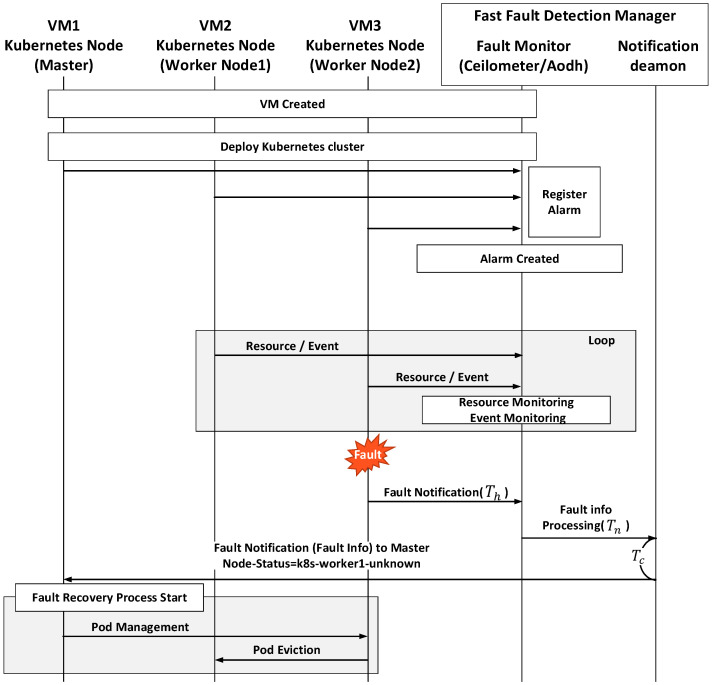
Fault detection and recovery procedures in the proposed architecture.

Figure 4 shows the fault detection and recovery procedures. When Aodh sends an alarm to the notification daemon, the alarm is delivered to the API server of the Kubernetes master and the API server forwards the alarm to the node controller to update the status of the faulty node from “Ready” to “Unknown.” This indicates that the node is not healthy and triggers the recovery process. The fault-detection time $T_{fd\text{-}proposed}$ in this procedure can be obtained as

(4) $$T_{fd\text{-}proposed}=T_{h}+T_{n}+T_{c}$$

where $T_{h}$ is the polling interval for checking the node status at the ceilometer, $T_{n}$ is the processing delay of the fault monitors, and $T_{c}$ is the processing delay of the notification daemon. The default value of $T_{h}$ is 300 ms, and $T_{n}$ and $T_{c}$ are the time it takes to transmit fault information to the Kubernetes master through REST API. After receiving the information from the ceilomter, it is converted based on the Kubernetes format and transmitted to the Kubernetes master through REST API. Format conversion and REST API processing are necessary only for faulty nodes. Comparing this fault-detection time with the previous one, we can see that delay is reduced by using direct fault detection at the VM level. 

## 4. System Validation

### 4.1. Implementation Environment and Methodology

For evaluation, a test environment was built using an OpenStack control node and two OpenStack compute nodes, as shown in Table 3. A Kubernetes cluster, consisting of 1 master node and 20 worker nodes, was deployed.

The proposed architecture was constructed, as shown in Figure 2 and Figure 3, and the existing architecture was constructed using Kubernetes’ optimized parameters. In this paper, we verify and analyze the performance of the existing Kubernetes fault-detection method and the proposed fault-detection method via experiments. First, the optimized parameters in the Kubernetes environment are confirmed, and then the performance of the optimized Kubernetes fault-detection method and that of the proposed method are compared with each other. Subsequently, we experimented with how the performance varies depending on the change in resource usage (CPU and network bandwidth). All experiments were conducted five times per scenario.

### 4.2. Evaluation

To verify the optimized Kubernetes parameters, VM fault-detection tests were conducted with various parameter sets, and the results are summarized in Table 4. In Table 4, we list the fault-detection times according to the parameter changes in the Kubernetes environment. According to the correlation between parameters, the experiment was conducted while changing the variables that affect the fault-detection time. Scenario 1 is the default value used in Kubernetes, and Scenario 2 is a parameter set with the minimum values. Scenarios 3 and 4 are the results of experiments with increasing $T_{g}$ values from those in Scenario 2. 

In Table 4, we list the fault-detection times according to the parameter changes in the Kubernetes environment. According to the correlation between parameters, the experiment was conducted, while changing the variables that affect the fault-detection time. Scenario 1 is the default value used in Kubernetes, and Scenario 2 is a parameter set with the minimum values. Scenarios 3 and 4 are the results of experiments with increasing $T_{g}$ values from those in Scenario 2. 

In Scenario 1, the default parameters defined in Kubernetes were used. It presents the worst results because the VM faults were only detected at a container level. In Scenario 2 and Scenario 3, $T_{u}$ and $T_{m}$ were set too short for the node update procedure to be completed within time $T_{g}$. In addition, if $T_{m}$ is not delivered within time $T_{u}$, it can be misjudged as a failure even if no failure occurred. Hence, it was difficult to correctly determine the VM defect. In Scenarios 4, the fault-detection time increased with $T_{g}$ because Kubernetes determined the fault after time $T_{g}$.

We compared these results with the results obtained using the proposed architecture. Fault detection time is the time taken from the time the fault occurs to the time when the master node receives the fault information. In the proposed architecture, it took an average of 0.84 seconds to detect the failure node. The fault detection time is composed of parameters of Equation 4. As shown in Table 4, the proposed architecture can detect faults about three times faster compared to using the optimized Kubernetes parameters (Scenario 4).

**Figure 5:**
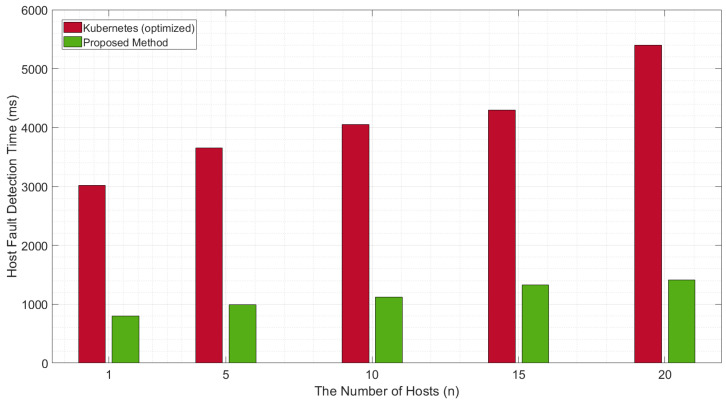
Comparison of the fault-detection time with increasing worker nodes.

We also checked the fault-detection time when the number of worker nodes was increased. Figure 5 shows that for the default Kubernetes, as the number of nodes increases, the time is taken to detect faults also increases because of the increased time required to check the node statuses. In contrast, the overall fault-detection time does not increase significantly for the proposed architecture, despite the increased time taken to poll the node states.

**Figure 6:**
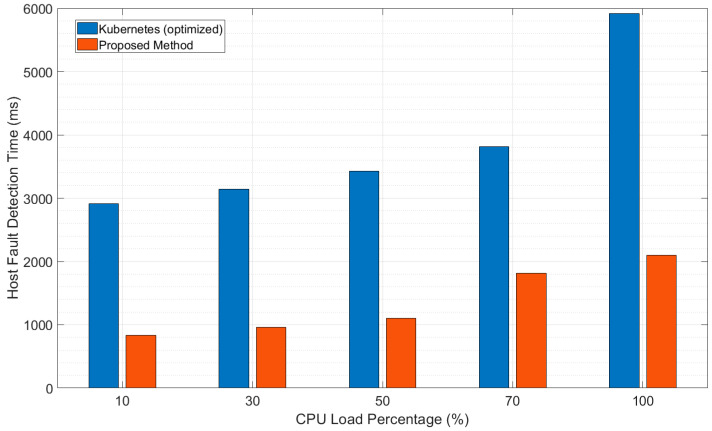
Comparison of fault-detection times with increasing CPU load.

**Figure 7:**
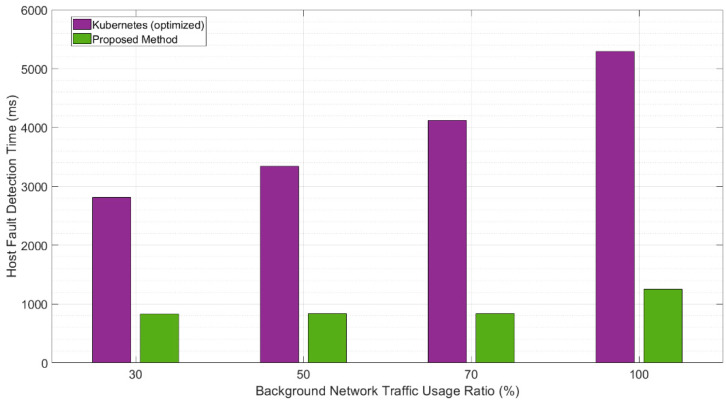
Comparison of fault-detection times with background network traffic.

Figure 6 and Figure 7 present the results when the fault-detection time was measured, while the CPU usage of the VM used for the Kubernetes master node and the traffic across the network between the master and worker nodes were increased, respectively. To increase the CPU usage of the master node, we generated additional workload using the stress-ng tool over the Kubernetes default process [26]. We experimented by generating a certain level of the workload on all CPUs assigned to the Master Node, while increasing the CPU usage until 100%. The CPU usage of the control node was also increased in the proposed architecture. From this experiment, we found that the fault-detection process was delayed as the CPU usage increased, but the delay was significantly lower for the proposed architecture compared to the usual architecture, as shown in Figure 6. 

To investigate the effect of the background network traffic between the master and worker nodes, we used the Iperf3 tool to generate background traffic [27]. The measurement was performed by increasing the bandwidth by up to 100% for each section, and the results are depicted in Figure 7. The increase in the background network traffic on Kubernetes delayed the checking and updating of the node-status information for fault diagnosis, and hence, increased the time for the entire fault-detection process. In contrast, because the proposed architecture does not update the node status continuously, but only when a fault occurs, the information is immediately transmitted to the master node without additional steps. Hence, the background network traffic does not significantly affect the performance, as shown in Figure 7.

All the above experiments confirm that the proposed architecture can detect faults faster than the existing method even when the size or overall resource usage in the Kubernetes cluster is increased. The proposed architecture can thus be used for IoT services requiring high infrastructure availability.

Discussion: In this paper, we proposed and implemented architecture to improve the fault detection performance of the infrastructure to improve the availability of container-based IoT services running on VMs. To do that, we analyzed the existing fault detection function based on Kubernetes, a representative container orchestrator. By analyzing the fault detection function, we confirmed the parameter setting for the optimized fault detection in the existing method. Although the optimized Kubernetes detected faults faster than the default settings, we found that the optimized settings increased the frequency of status updates and resulted in increased network and CPU resource usage. Further, the node faults were occasionally misjudged. After that, we designed an architecture for the integrated and automated fault management of VM and container manager.

Through experimentation, in the VM environment, fault information is checked from the hypervisor every 300 ms, and when a failure occurs, it is delivered to the function responsible for managing the fault immediately, so that failure can be detected faster than in the existing container environment. On the other hand, it is confirmed that fault detection in a container environment needs additional time to check and update fault information. Even in the proposed architecture, format change and transmission process are required when transmitting fault information, but this is a process required only for the node in which the failure occurred, and It was confirmed that the time of process did not significantly affect the overall performance.

In addition, we confirmed that the proposed architecture can detect faults faster than the optimized Kubernetes without significant changes in the performance and resource usage even when the number of nodes was increased. Through the experimental results and structural features (event-driven message delivery), it was confirmed that the proposed architecture has higher scalability compared with the existing architecture. However, similarities exist in limitations between our architecture and the existing one regarding the use of one controller, and in order to use multiple controllers, necessitates discussion of a new architecture.

Further experiments to investigate the relationship between the resource usage of background IoT applications and fault-detection performance revealed that the performance of Kubernetes fault detection methods declined as the resource usage increased. Although the overall performance of the proposed architecture also decreased, faults could still be detected up to three times faster than in the case of the optimized Kubernetes parameter settings. In the same way, performance measurements were performed while increasing the RAM resource usage of the background application, but the increase in RAM usage affected the CPU usage, and it was confirmed that the results were similar.

Through the experimental results, it was confirmed that the performance of the proposed architecture was not significantly affected by resource usage. The most influential factor of fault tolerance in the proposed system is resource usage, so it was confirmed that the proposed architecture offers better fault tolerance.

Our first contribution point is automation: When configuring a node, it provides a function to automatically register a fault alarm, and if a problem occurs during operation, the fault information is sent directly to the manager. Through this, each manager can get the notification immediately when the node fails. The second contribution is VM and container manager integration. In the existing method, the fault is judged by receiving data from the agent of each node. However, as shown in Table 4, there is a limitation in improving the fault detection time because of limitations in parameter settings. However, in the proposed architecture, the failure detection time is improved by passing the node’s failure information directly to the container manager. Through the proposed architecture, the performance of the fault detection has been improved threefold. In addition, we also confirmed that the proposed architecture is suitable for environments where resource usage is constantly changing, i.e., dynamic, such as a cloud environment. 

However, we observed that for the proposed architecture, the performance gradually decreased as the number of nodes increased or resource usage changed. In other words, it is expected that further research is needed to mitigate the performance degradation. Likewise, for a multi-cluster environment, it is necessary to additionally consider a “collecting the node status” method and a communication method between the multi-container managers. The existing architecture is where multiple sites are managed by a single controller, but if multiple controllers are to be used, it is expected that additional research on the distribution of management functions by site and appropriate data collection methods will be required.

## 5. Conclusions

In this study, we analyzed the fault-detection function of the container infrastructure used as the infrastructure of an IoT cloud and proposed an integrated infrastructure management architecture to realize fast fault detection. Based on the proposed architecture, we implemented a fast-fault-detection function that can provide high availability for mission-critical services, such as V2V communication and real-time services. 

The proposed architecture can detect faults faster than the state of the art even when the IoT cloud size or background application resource usage increases. Moreover, it can improve the management function for container clusters currently running on various cloud provider’s infrastructures. By applying the proposed architecture, it is possible to detect fault using fault information of the infrastructure of VMs in which various IoT services operate, which will be useful in terms of service management.

Future research will study the optimization of recovery methods for IoT applications in a multi-cloud environment. Accordingly, it is necessary to study the distributed cloud management architecture and how to apply the monitoring and information delivery functions proposed in this paper. In addition, additional monitoring methods should be considered for precise monitoring in a distributed cloud environment. It is expected that this will enable efficient management of IoT services deployed in distributed clouds, such as Edge Cloud.

## Figures and Tables

**Table 1:** Fault management methods in Kubernetes.

Level.	Fault Type	Manager	Fault Detection Method	Fault Recovery Method
Application	Application (service)	Health Check Daemon	TCP/HTTP/CMD	Restart
		External monitoring function	External monitoring matrix	External tool function
Infrastructure	Container	Kubelet (cAdvisor)	Process list	Restart
	Pod	Kubelet (cAdvisor)	Process list	Respawn
	Node	Node controller	Message (Node to Kubelet)	Notification
		External monitoring function	External monitoring matrix	External tool function

**Table 2:** Parameters related to the fault-detection process.

Parameter	Meaning
$T_{u}$	The node-status-update-frequency (duration: seconds)
$T_{m}$	The node-monitor-period (seconds)
$T_{g}$	The node-monitor-grace-period (seconds)
$T_{e}$	The pod-eviction-timeout (seconds)
$T_{f}$	The time interval between the fault occurrence point and the grace period endpoint (seconds)

**Table 3:** Implementation specifications.

Entity	Condition	Version
Physical Server (3)	Controller Node (1) Intel^®^ Xeon^®^ processor D-1557, Single-socket FCBGA 1667; 12-core, 24 threads RAM: 64 GB Disk space: 1TB Compute Node (2) Intel^®^ Xeon^®^ processor D-1557, Single-socket FCBGA 1667; 12-core, 24 threads, RAM: 64 GB Disk space: 1TB	
Cloud OS	OpenStack stable	Stein
Container infrastructure	Kubernetes (Master Node: 1EA/Worker Node: 20EA)	1.17.1

**Table 4:** Experimental results for Kubernetes parameter optimization.

	Parameter	$T_{u}$	$T_{g}$	$T_{m}$	Fault-Detection Time (FDT)
Scenario					
Scenario 1		10 s	40 s	5 s	MIN = 40 s
Scenario 2		1 s	1 s	1 s	Error
Scenario 3		1 s	2 s	1 s	Error
Scenario 4		1 s	3 s	1 s	MIN = 3 s
